# Lipid accumulation product is a powerful tool to predict non-alcoholic fatty liver disease in Chinese adults

**DOI:** 10.1186/s12986-017-0206-2

**Published:** 2017-08-01

**Authors:** Haijiang Dai, Weijun Wang, Ruifang Chen, Zhiheng Chen, Yao Lu, Hong Yuan

**Affiliations:** 1 Center of Clinical Pharmacology, the Third Xiangya Hospital, Central South University, 138 Tong-Zi-Po Road, Changsha, Hunan 410013 People’s Republic of China; 2 Center of Health Management, the Third Xiangya Hospital, Central South University, Changsha, Hunan Province 410013 People’s Republic of China; 3 Division of Gastroenterology, Union Hospital, Huazhong University of Science and Technology, Wuhan, Hubei Province 430022 People’s Republic of China

**Keywords:** LAP, NAFLD, Severity, General population

## Abstract

**Background:**

Non-alcoholic fatty liver disease (NAFLD), recognized as the liver manifestation of metabolic syndrome, is highly prevalent in the general population. Recent studies suggest that lipid accumulation product is significantly associated with metabolic abnormalities. The aim of this study was to assess the accuracy of lipid accumulation product (LAP) as an effective screening tool for diagnosing NAFLD in the general population.

**Methods:**

A total of 40,459 subjects aged ≥18 years were enrolled in this cross-sectional study. LAP was calculated as [waist circumference (cm) – 65] × triglyceride concentration (mmol//L) in men and [waist circumference (cm) – 58] × triglyceride concentration (mmol/L) in women. Multiple logistic regression and receiver operating characteristic (ROC) analyses were performed.

**Results:**

According to multiple logistic regression analyses, LAP was significantly associated with a higher prevalence and severity of NAFLD in both men and women. When assessed using ROC curve analyses, LAP exhibited high diagnostic accuracy for identifying NAFLD, and the areas under the curves (AUC) in men and women were 0.843 (95% CI 0.837, 0.849) and 0.887 (95% CI 0.882, 0.892), respectively. After further analyzed in different age groups, the diagnostic accuracy of LAP was found to be significantly better in younger age groups (aged 18-34 for men; aged 18-34 and 35-44 years for women) for both sexes.

**Conclusions:**

LAP is significantly associated with the presence and severity of NAFLD, and has a high diagnostic accuracy for identifying NAFLD in the general population. The diagnostic accuracy of LAP was especially high among younger age groups.

**Electronic supplementary material:**

The online version of this article (doi:10.1186/s12986-017-0206-2) contains supplementary material, which is available to authorized users.

## Background

Non-alcoholic fatty liver disease (NAFLD), recognized as a major public health concern, is highly prevalent in the general population [1]. In China, NAFLD affects over a quarter of the general population, and its prevalence is rapidly increasing as a result of considerable lifestyle changes and the aging of the population [2, 3]. NAFLD can progress to serious complications such as cirrhosis, hepatocellular carcinoma and death. Indeed, it was reported that NAFLD has become the leading cause of hepatocellular carcinoma [4] and the second most common cause of liver transplantation [5] in the United States. In addition to these hepatic complications, numerous epidemiological studies have also demonstrated that NAFLD patients have an increased risk of diabetes, metabolic syndrome and cardiovascular disease [6–9].

Although NAFLD causes tremendous economic and clinical burden, the notion of conducting widespread screening for NAFLD in the general population remains controversial [10]. Because liver biopsy is an invasive technique that has been associated with some morbidity and very rare mortality risk, it is not a realistic screening test. Liver ultrasound is potentially more safe but it is still expensive and cumbersome as a screening test [10, 11]. Thus, it would be useful if a simple and inexpensive index is available for accurate diagnosis of individuals at risk of NAFLD in clinical settings.

Lipid accumulation product (LAP), which is calculated as a combination of waist circumference (WC) and fasting plasma triglyceride (TG) levels, has been proposed as an alternative measure of excessive lipid accumulation [12]. Recently, a growing number of studies have shown that LAP is a powerful marker for diabetes [13], metabolic syndrome [14, 15], and insulin resistance [16] in the general population and associated with risk of cardiovascular diseases [17–19]. Given the close associations between diabetes, metabolic syndrome, insulin resistance, and hepatic steatosis [20], these findings inspired us to explore whether LAP could also be a powerful index for identifying NAFLD. In this study, we performed a cross-sectional study to test the accuracy of LAP as a marker for NAFLD in the general population.

## Methods

### Study population

The Health Management Center of the Third Xiangya Hospital is one of China’s largest examination centers, and it mainly services people from hundreds of institutions in Changsha. From January 2014 to December 2014, a cross-sectional study was conducted based on the population who participated in an annual physical examination in the center. As a result, 84,572 subjects aged ≥18 years agreed to be included and were enrolled in this cross-sectional study. The study protocol was approved by the Medical Ethics Committee of the Third Xiangya Hospital. All experiments in this study were performed according to the guidelines from the Helsinki Declaration and informed written consent was obtained from all subjects.

In the present study, we included 51,070 subjects in whom an abdominal ultrasonography examination was performed and for whom we obtained complete information regarding alcohol consumption. The following exclusion criteria were as applied: 1) excessive alcohol drinking, which was defined as an average weekly consumption of alcohol ≥140 g for men and ≥70 g for women (*n* = 6499) [21, 22]; 2) viral hepatitis, schistosomiasis liver disease or other chronic liver diseases (*n* = 1953); 3) a history of taking steatogenic medications, such as corticosteroids, methotrexate, etc. (*n* = 43); 3) no available data on TG or WC, or those who were pregnant (*n* = 1472); 5) a history of taking lipid-lowering medications (*n* = 644). After applying these exclusions, a total of 40,459 subjects were ultimately screened and deemed eligible for this study.

### Data collection and measurements

All subjects were interviewed by trained interviewers using pretested questionnaires to collect information on age, sex, alcohol consumption, cigarette smoking, physical activity, education level, and medical history. For alcohol consumption, information in three aspects was collected: type of alcoholic beverages, drinking frequency per week and the usual amount per time. Subjects with an average weekly consumption of alcohol ≥140 g for men and ≥70 g for women were considered as excessive alcohol consumption [21, 23]. Cigarette smoking was recorded as daily (at least one cigarette/day), occasional (less than one cigarette/day), former (having quit for at least 6 months), or never smoking. For the analyses, only two categories were considered: current smoking (daily and occasional smoking) and non-smoking (never and former smoking) [24]. Physical activity was evaluated based on responses to questions regarding the frequency of physical activity during leisure time and scored as inactive (0-2 times per week), moderate (3-5 times per week), active (≥6 times per week) [25].

Weight and height (with outdoor clothing and shoes removed) were measured in a standing position using calibrated weighing scales, and body mass index (BMI) was calculated as weight in kilograms divided by height in meters squared. WC was measured at the umbilical level using an un-stretched tape measure and without applying any pressure to the body surface. Blood pressure (BP) was measured using a corrected mercury sphygmomanometer in the right arm. Two readings were obtained for both SBP and DBP after a 10 min rest in a sitting position, with a 30 s interval. The mean of the two readings was considered the subject’s BP. If the two readings differed by >5 mmHg, BP was re-measured, and the subject’s BP was finally calculated as the average of the three readings.

Blood samples were collected from the antecubital vein in the morning after an overnight fast and then transferred into EDTA-containing vacuum tubes. Blood samples were then stored at -20 °C until analyzed. Concentrations of fasting blood glucose (FBG), TG, total cholesterol (TC), high-density lipoprotein cholesterol (HDL-C), serum uric acid and creatinine were determined by enzymatic colorimetric assay. Concentration of alanine transaminase (ALT) was measured by continuously ultraviolet monitoring method. Concentration of total bilirubin (TBIL) was measured by vanadate oxidase method. All blood samples were tested using an auto-analyzer (Hitachi 7600-110; Hitachi, Tokyo, Japan) at the central laboratory of the Third Xiangya Hospital. The intra-assay coefficients of variation for FBG, TG, TC, HDL-C, ALT, TBIL, serum uric acid and creatinine were 2.24, 4.38, 3.23, 1.73, 3.91, 2.01, 2.36, and 4.47%, respectively.

### Assessment of lipid accumulation product

LAP was calculated using the formula [WC (cm) – 65] × TG concentration (mmol/L) for men, and [WC (cm) – 58] × TG concentration (mmol/L) for women [12, 17, 26]. To avoid obtaining non-positive values for LAP, in men, any WC values that were 65 cm or less were revised upward to 66.0 cm (*n* = 180), and in women, those that were 58 cm or less were revised upward to 59.0 cm (*n* = 101), as suggested by Kahn [12].

### Assessment of non-alcoholic fatty liver disease

A diagnosis of NAFLD was based on the presence of hepatic steatosis on liver ultrasonography that was not the result of acute or chronic liver diseases and was not related to secondary hepatic fat accumulation, including excessive alcohol consumption and the use of steatogenic medication [10, 27]. Liver ultrasonography was performed by experienced and trained radiologists who were blinded to the subjects’ clinical diagnosis and biochemical tests. The ultrasonographic criteria of hepatic steatosis included diffusely increased liver near-field ultrasound echo (‘bright liver’), liver echo greater than kidney; vascular blurring and the gradual attenuation of a far-field ultrasound echo. Subjects with at least two of the abnormal findings listed above were diagnosed with hepatic steatosis [21, 27]. The presence and severity of hepatic steatosis was recorded using a numbered scoring system (1 = absent; 2 = mild; 3 = moderate; and 4 = severe) [28].

### Statistical analysis

Basic characteristics of the study subjects were summarized as numbers with percentages for categorical variables and as the mean ± standard deviation (SD) for continuous variables. Variables that displayed a skewed distribution (age, FBG, ALT, TBIL, TG and LAP) were log-transformed to normal before analysis. Comparisons of basic characteristics by NAFLD status (with/without) were assessed using Pearson’s χ2 test for categorical variables and Student’s t-tests for continuous variables.

To obtain a deeper understanding of the relationship between LAP levels and the prevalence of NAFLD, we next divided the study population into 4 groups according to LAP quartiles (Q1: <10.53, Q2: 10.53 to <20.59, Q3: 20.59 to <38.48, Q4: ≥38.48). Comparisons of the prevalence of NAFLD between different groups were assessed using Pearson’s χ2 test. Adjusted odds ratios (ORs) with 95% confidence intervals (CIs) were calculated using logistic regression analysis to determine the risk of NAFLD in each LAP quartile and using the lowest quartile as the reference. The area under the curve (AUC) was assessed using receiver operating characteristics (ROC) to evaluate the strength of the association between LAP levels and NAFLD. The best possible cut-off point was defined as the highest Youden Index [(specificity + sensibility) − 1] [29]. Finally, differences between AUCs were tested using a nonparametric approach [30].

All analyses were performed using IBM SPSS version 22.0 (IBM Corp., Armonk, NY, USA) and STATA version 14.0 (Stata Corp., College Station, TX, USA), and two-tailed *P* values of <0.05 were considered statistically significant.

## Results

### Characteristics of the study subjects

During the study, a total of 40,459 Chinese participants (18,336 men and 22,123 women; age: 43.7 ± 14.1 years, range: 18-94 years) were included. Among these subjects, 8070 (44.0%) men and 4080 (18.4%) women were diagnosed with NAFLD. Table 1 depicts the clinical and biochemical characteristics of the subjects according to NAFLD status. For both sexes, patients with NAFLD were older and had higher LAP, BMI, WC, SBP, DBP, FBG, platelet count, ALT, uric acid, TG, TC, and reduced TBIL, HDL-C levels than those observed in the subjects without NAFLD. Meanwhile, the patients with NAFLD were more likely to take antihypertensive and hypoglycemic drugs in both sexes. However, there was a significantly higher prevalence of current smoker in men but not in women. Additionally, the frequencies of physical activity and levels of creatinine were significantly different in women but not in men.

**Table 1:** Characteristics of the study subjects with and without non-alcoholic fatty liver disease

	Men		*P* value	Women		*P* value
	Non-NAFLD	NAFLD		Non-NAFLD	NAFLD	
*N*	10,266	8070		18,043	4080	
LAP^a^	23.7 ± 22.0	62.4 ± 59.7	<0.001	16.6 ± 15.5	49.4 ± 41.2	<0.001
Age (years)^a^	44.1 ± 15.8	46.8 ± 13.7	<0.001	40.3 ± 12.4	51.8 ± 12.1	<0.001
BMI (kg/m^2^)	23.1 ± 2.6	26.7 ± 2.6	<0.001	21.7 ± 2.4	25.9 ± 2.9	<0.001
WC (cm)	80.8 ± 7.2	90.5 ± 6.9	<0.001	73.2 ± 7.0	84.8 ± 7.4	<0.001
Current smoker, %	4292 (42.3)	3599 (45.1)	<0.001	669 (3.8)	152 (3.8)	0.871
Physical activity			0.135			<0.001
Inactive, %	5947 (60.9)	4587 (59.5)		11,127 (67.2)	1938 (53.5)	
Moderate, %	2465 (25.2)	2041 (26.5)		3931 (23.7)	1035 (28.6)	
Active, %	1355 (13.9)	1081 (14.0)		1510 (9.1)	647 (17.9)	
Education			0.050			<0.001
Illiteracy/Primary, %	139 (1.4)	91 (1.2)		282 (1.6)	201 (5.2)	
Middle school, %	1660 (16.7)	1400 (17.9)		2977 (17.1)	1345 (35.1)	
College or higher, %	8121 (81.9)	6316 (80.9)		14,119 (81.2)	2287 (59.7)	
Drinking, %	3613 (35.2)	3038 (37.6)	0.001	814 (4.5)	129 (3.2)	<0.001
SBP (mmHg)	123.9 ± 14.0	129.3 ± 14.5	<0.001	114.6 ± 14.0	127.5 ± 17.5	<0.001
DBP (mmHg)	76.0 ± 9.5	81.2 ± 10.3	<0.001	70.3 ± 9.3	77.0 ± 10.7	<0.001
FBG (mmol/L)^a^	5.2 ± 1.0	5.7 ± 1.6	<0.001	5.0 ± 0.7	5.7 ± 1.6	<0.001
Platelet count (10^9^/L)	208.7 ± 50.5	215.9 ± 51.8	<0.001	222.3 ± 53.2	232.0 ± 57.4	<0.001
ALT (U/L)^a^	26.0 ± 17.3	40.2 ± 26.0	<0.001	17.5 ± 11.5	26.6 ± 18.6	<0.001
TBIL (μmol/L)^a^	16.6 ± 5.6	15.8 ± 5.4	<0.001	15.2 ± 4.7	14.2 ± 4.2	<0.001
Uric acid (μmol/L)	325.9 ± 74.6	368.4 ± 82.7	<0.001	220.3 ± 59.0	274.1 ± 71.0	<0.001
TG (mmol/L)^a^	1.4 ± 0.9	2.4 ± 2.1	<0.001	1.0 ± 0.6	1.8 ± 1.4	<0.001
TC (mmol/L)	4.9 ± 0.9	5.3 ± 1.0	<0.001	4.9 ± 0.9	5.4 ± 1.0	<0.001
HDL-C (mmol/L)	1.5 ± 0.4	1.3 ± 0.3	<0.001	1.9 ± 0.4	1.6 ± 0.3	<0.001
Creatinine (μmol/L)	78.3 ± 19.6	77.8 ± 15.6	0.082	54.3 ± 12.1	55.3 ± 11.0	<0.001
Use of antihypertensive, %	807 (7.9)	1153 (14.3)	<0.001	675 (3.7)	742 (18.2)	<0.001
Use of hypoglycemic, %	253 (2.5)	378 (4.7)	<0.001	134 (0.7)	175 (4.3)	<0.001

*Abbreviations*: *LAP* lipid accumulation product, *BMI* body mass index, *WC* waist circumference, *SBP* systolic blood pressure, *DBP* diastolic blood pressure, *FBG* fasting blood glucose, *ALT* alanine transaminase, *TBIL* total bilirubin, *TG* triglyceride, *TC* total cholesterol, *HDL-C* high-density lipoprotein cholesterol

^a^variables were log-transformed before analysis

### Association between lipid accumulation product and the presence of non-alcoholic fatty liver disease

To more effectively explore the relationship between LAP and the prevalence of NAFLD, we divided the subjects into four groups according to their LAP quartiles. As a result, the prevalence of NAFLD progressively increased in the higher LAP quartile groups in both men (2.4, 16.2, 43.4, and 76.1%, respectively) and women (0.7, 7.4, 29.1, and 63.2%, respectively).

Table 2 shows the multiple adjusted association between quartiles of LAP and NAFLD. In men, after adjusting for age, the OR for NAFLD in the comparison between the highest and lowest quartiles of LAP was 122.50 (95% CI 94.98, 157.99). After further adjusting for current smoker, physical activity, education, ALT, TBIL, SBP, DBP, FBG, uric acid, TC, HDL-C, creatinine, the use of antihypertensive and hypoglycemic, the risk for the prevalence of NAFLD was lower and the OR for the comparison between the highest and lowest quartile of LAP was 37.22 (95% CI 28.05, 49.38). However, among women, a relatively stronger association was found between quartiles of LAP and NAFLD was found. In multivariate-adjusted model 3, the OR for NAFLD in the highest compared with the lowest quartile of LAP was 48.06 (95% CI 34.36, 67.23). Similar results were also observed when LAP was considered as a continuous exposure variable (per SD increment, Table 2).

**Table 2:** Odds ratios and 95% confidence intervals for non-alcoholic fatty liver disease according to quartiles of lipid accumulation product, per SD increase and the cut-off

	Quartiles of LAP				Per SD increment	Cut-off
	Q1	Q2	Q3	Q4		
Men						
*N*	2634	3741	5211	6750		8768
Model 1	1 (Ref)	7.42 (5.70, 9.65)	29.02 (22.51, 37.43)	122.50 (94.98, 157.99)	7.50 (7.04, 8.00)	10.21 (9.53, 10.93)
Model 2	1 (Ref)	5.71 (4.35, 7.49)	19.66 (15.12, 25.56)	67.02 (51.49, 87.24)	6.10 (5.69, 6.54)	7.18 (6.65, 7.75)
Model 3	1 (Ref)	4.66 (3.53, 6.16)	13.41 (10.21, 17.62)	36.17 (27.26, 48.00)	5.28 (4.84, 5.75)	4.28 (3.91, 4.69)
Women						
*N*	7477	6378	4900	3368		7120
Model 1	1 (Ref)	9.20 (6.89, 12.30)	39.62 (29.84, 52.59)	152.00(114.16, 202.38)	8.77 (8.11, 9.48)	11.96(10.90,13.13)
Model 2	1 (Ref)	8.43 (6.15, 11.56)	32.37 (23.76, 44.09)	105.92 (77.41, 144.93)	7.64 (7.00, 8.35)	9.49 (8.55, 10.53)
Model 3	1 (Ref)	6.67 (4.85, 9.18)	19.28 (14.02, 26.52)	46.22 (33.04, 64.66)	5.76 (5.16, 6.42)	4.70 (4.16, 5.30)

Model 1: adjusted for age^a^;

Model 2: further adjusted for current smoker, physical activity, education, drinking, alanine transaminase^a^, total bilirubin^a^;

Model 3: further adjusted for systolic blood pressure, diastolic blood pressure, fasting blood glucose^a^, platelet count, uric acid, total cholesterol, high-density lipoprotein cholesterol, creatinine, use of antihypertensive and hypoglycemic

^a^: variables were log-transformed before analysis

Per SD increment: per SD increment of log lipid accumulation product

Cut-off: the OR (95% CI) for NAFLD in the comparison between above and below the cut-off value of LAP

### Association between lipid accumulation product and the severity of non-alcoholic fatty liver disease

Table 3 shows the multiple adjusted association between LAP and the severity of NAFLD. In men, after adjusting for age, current smoker, physical activity, education, ALT, TBIL, SBP, DBP, FBG, uric acid, TC, HDL-C, creatinine, the use of antihypertensive and hypoglycemic, per SD increment of log LAP was significantly associated with increased likelihood (OR 4.34, 95% CI 3.96, 4.76; *P* < 0.001) of having mild NAFLD. The association of LAP with moderate and severe NAFLD were even more pronounced (OR 7.07, 95% CI 6.23, 8.02; *P* < 0.001 and OR 17.90, 95% CI 10.57, 30.30; *P* < 0.001). Tests of heterogeneity demonstrated that the effect of LAP on different grades of NAFLD was significantly different from each other (moderate vs mild, heterogeneity *P* < 0.001; severe vs mild, heterogeneity *P* < 0.001; severe vs moderate, heterogeneity *P* = 0.001). Similarly, the effect of LAP on different grades of NAFLD were consistent in women (Table 3).

**Table 3:** Association between LAP levels and the severity of non-alcoholic fatty liver disease in subjects

	Grade	*N*	Per SD increment of LAP^a^	*P* value	*P* value^#^	*P* value^##^
Men	None	10,266	1 (Ref)	─	─	─
	Mild	4621	4.32 (3.94, 4.74)	<0.001	─	─
	Moderate	3310	7.07 (6.23, 8.03)	<0.001	<0.001	─
	Severe	139	19.61 (11.48, 33.51)	<0.001	<0.001	<0.001
Women	None	18,043	1 (Ref)	─	─	─
	Mild	2497	4.90 (4.35, 5.52)	<0.001	─	─
	Moderate	1530	7.72 (6.46, 9.23)	<0.001	<0.001	─
	Severe	53	23.84 (9.81, 57.90)	<0.001	<0.001	0.015

Adjusted for age^a^, current smoker, physical activity, education, drinking, alanine transaminase^a^, total bilirubin^a^, systolic blood pressure, diastolic blood pressure, fasting blood glucose^a^, platelet count, uric acid, total cholesterol, high-density lipoprotein cholesterol, creatinine, use of antihypertensive and hypoglycemic

^a^: variables were log-transformed before analysis

^#^Test of heterogeneity for the effect of LAP levels on the severity of non-alcoholic fatty liver disease: moderate vs mild, severe vs mild

^##^Test of heterogeneity for the effect of LAP levels on the severity of non-alcoholic fatty liver disease: severe vs moderate

### Accuracy of lipid accumulation product for diagnosing non-alcoholic fatty liver disease

**Fig. 1:**
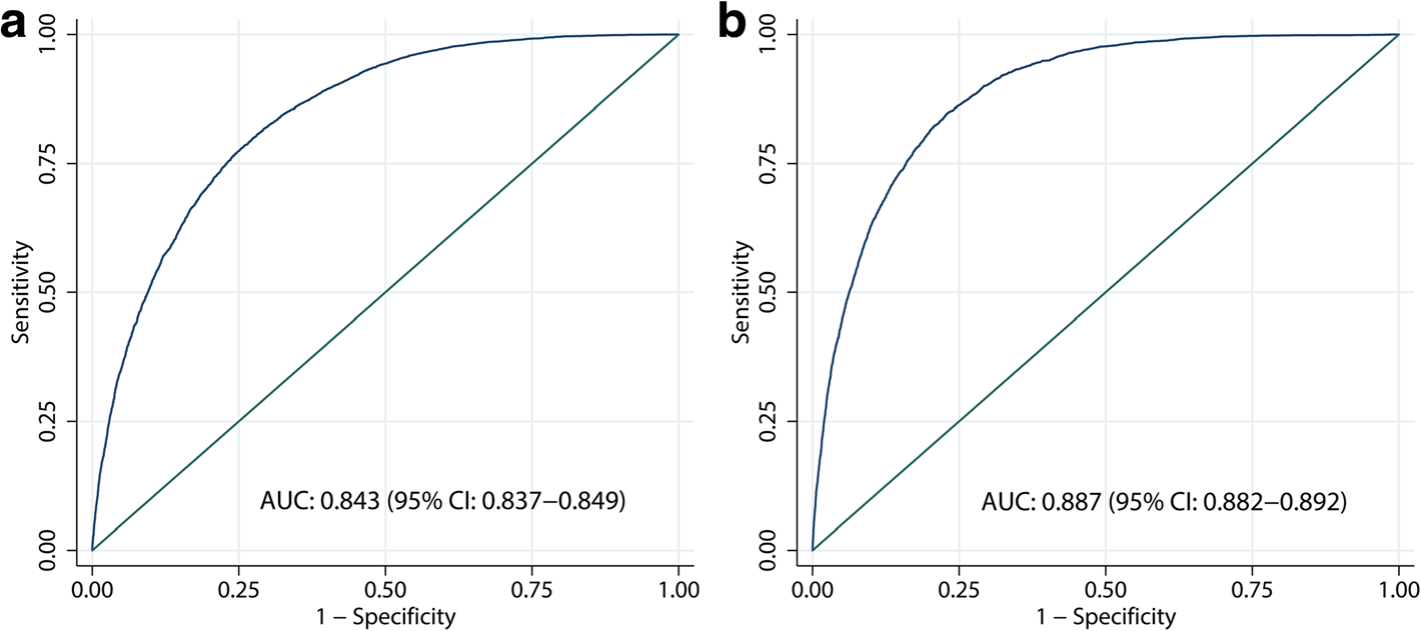
ROC analysis of lipid accumulation product for the prediction of non-alcoholic fatty liver disease in men (**a**) and women (**b**). **a** The areas under the ROC curve for the lipid accumulation product as a predictor of non-alcoholic fatty liver disease in men was 0.843 (95% CI 0.837, 0.849). **b** The areas under the ROC curve for the lipid accumulation product as a predictor of non-alcoholic fatty liver disease in women was 0.887 (95% CI 0.882, 0.892)

Using the ROC curve analyses, we evaluated the accuracy of LAP for diagnosing NAFLD in both men and women. As shown in Fig. 1, LAP exhibited high diagnostic accuracy for identifying NAFLD, and the areas under the curves (AUC) in men and women were 0.843 (95% CI 0.837, 0.849) and 0.887 (95% CI 0.882, 0.892), respectively. The identified cut-off values for LAP in men and women were 30.5 (sensitivity: 77%, specificity: 75%) and 23.0 (sensitivity: 82%, specificity: 79%), respectively.

Multivariate binary logistic regression analyses (Table 2) showed that the proposed LAP cut-off values were significantly associated with NAFLD in both men and women. In the multivariate-adjusted model 3, the OR for LAP above the cut-off value compared with that below the cut-off value was 4.32 (95% CI 3.94, 4.73) in men and 4.84 (95% CI 4.29, 5.46) in women.

### Assessment of the predictive accuracy of lipid accumulation product in different age groups

Table 4 shows the AUC for LAP as a predictor of NAFLD in different age groups. For both sexes, the diagnostic accuracy of LAP was significantly better in younger age groups (aged 18-34 for men; aged 18-34 and 35-44 years for women) than that in those aged ≥75 years. However, the difference in AUC for LAP between other age groups and those aged ≥75 years was not statistically significant in both men (*P* = 0.346, 0.511, 0.673, 0.103, respectively) and women (*P* = 0.090, 0.381, 0.963, respectively).Similar results were also found in normal weight groups﻿ (BMI≤22.9 kg/m^2^ for ﻿both men and women, Additional file 1).

**Table 4:** Areas under the ROC curves for lipid accumulation product as a predictor of non-alcoholic fatty liver disease in subjects of different ages

Age, years	Men		*P* value	*P* value^#^	Women		*P* value	*P* value^#^
	*N*	AUC (95% CI)			*N*	AUC (95% CI)		
18 ~ 34	5207	0.880 (0.871, 0.889)	<0.001	<0.001	7436	0.919 (0.906, 0.931)	<0.001	<0.001
35 ~ 44	4643	0.837 (0.825, 0.848)	<0.001	0.346	5634	0.863 (0.849, 0.877)	<0.001	0.002
45 ~ 54	3919	0.813 (0.800, 0.827)	<0.001	0.511	4987	0.829 (0.817, 0.841)	<0.001	0.090
55 ~ 64	2213	0.817 (0.799, 0.834)	<0.001	0.673	2667	0.809 (0.793, 0.825)	<0.001	0.381
65 ~ 74	1379	0.794 (0.771, 0.818)	<0.001	0.103	1037	0.789 (0.761, 0.816)	<0.001	0.963
75 ~	975	0.823 (0.797, 0.849)	<0.001	─	362	0.787 (0.741, 0.833)	<0.001	─

^#^comparison of AUC, with those aged 75 years or older serving as the reference

## Discussion

In this cross-sectional study, we found that LAP was significantly associated with a higher prevalence and severity of NAFLD in both men and women. When assessed using ROC curve analyses, LAP exhibited high diagnostic accuracy for identifying NAFLD. The diagnostic accuracy of LAP was especially high in younger age groups.

Recently, an increasing number of studies have focused on NAFLD as it is increasingly prevalent, with reported prevalence from 27 to 34% in North America [31–33]. In Europe, the prevalence of NAFLD varies by region with a reported low prevalence rate of 8% in Romania to a reportedly high prevalence of 45% in Greece [34–37]. The prevalence of NAFLD in Asia was also widely reported from 15 to 38% [38]. In our study, we found that the prevalence of NAFLD was 30.0%, with an obviously higher prevalence was shown in men. The LAP, which was introduced by Kahn [12], is a novel index of excessive lipid accumulation. Over the past decade, a growing body of research have found a significant correlation between LAP and cardiometabolic risk factors. According to a cross-sectional study that involved 8671 subjects, LAP was found to be a strong predictor of diabetes [13]. Taverna et al. [15] found that LAP had the highest diagnostic accuracy for metabolic syndrome, with an AUC of 0.91 and 0.90 in males and females. This result was similar to those reported by Xiang et al. [39] and Chan et al. [40]. Moreover, Xia et al. [16] also found that LAP was a powerful index for recognizing insulin resistance in non-diabetic individuals. These compelling findings raise the question whether LAP is a powerful maker for the identification of NAFLD.

In addition, both abdominal obesity and elevated TG levels play an important role in the pathogenesis of NAFLD. In abdominal obesity, visceral adipocytes produce several adipocytokines, such as adiponectin, resistin, and leptin, which increase insulin resistance [41]. Excessive adipocytes are also associated with a chronic inflammatory response, which is characterized by abnormal cytokine production and the activation of pro-inflammatory signaling pathways [42]. These pathophysiological changes might promote the development of NAFLD [42]. Moreover, NAFLD is characterized by the accumulation of TG and other fats in liver cells. The role of elevated TG levels in NAFLD has been supported by the reduction in hepatic steatosis after TG-lowering treatment with omega-3 fatty acids [43, 44]. Accordingly, it is plausible that the LAP, which is a combination of WC and fasting TG, is significantly associated with NAFLD. However, few studies have so far investigated the association between LAP and NAFLD, and the existing studies have been conducted primarily in subjects with chronic diseases, such as HIV infection [45], hepatitis B virus infection [46], and polycystic ovary syndrome [47], with inconsistent results. Whether LAP is an effective maker for NAFLD that could be widely applied in general Chinese population remains unknown. In this study, we demonstrate that LAP is significantly associated with the presence and severity of NAFLD, and has a high diagnostic accuracy for identifying NAFLD in a general Chinese population.

NAFLD is a chronic progressive disease, and its progression usually take years or even decades. Increasing the early detection and prevention of NAFLD, especially among young people, could effectively reduce the burden of cirrhosis and hepatocellular carcinoma. This characteristic of NAFLD prompted us to investigate the accuracy of LAP in different age groups. Interestingly, we found that the diagnostic accuracy of LAP was significantly better in younger age groups, in which we found an AUC of approximately 0.9 in both young men and women (aged <35 years). Our results suggest that LAP is a powerful marker that could be used to screen NAFLD, especially in younger age groups. We consider that the close association between LAP and NAFLD was rarely affected by other chronic conditions or the ability of lipid metabolism in younger age groups. Further studies are required to validate these findings and explain the effect of age on the diagnostic accuracy of LAP.

There are some limitations to the present study. First, our study was mainly conducted in a Chinese Han population. Further studies are needed to determine whether our results are applicable to other ethnic groups. Second, the information on viral hepatitis testing was prone to lacking, which may affect the exclusion of subjects. Third, some biochemical variables were not available for calculating other indices such as FLI in our study. Further studies are needed to compare the discriminative ability for NAFLD between LAP and other indices. Fourth, the presence of hepatic steatosis was assessed using ultrasonography rather than liver biopsy pathology. However, ultrasonography is widely accepted as a measure in population-based studies because of its safety, economical advantage and reasonable accuracy [10, 48].

## Conclusion

In conclusion, our study found that LAP is significantly associated with the presence and severity of NAFLD, and has a high diagnostic accuracy for identifying NAFLD in the general population. The diagnostic accuracy of LAP was especially high among younger age groups. People, especially young people, with high levels of LAP deserve particular attention for NAFLD.

## Additional file

Additional file 1: Table S1. Areas under the ROC curves for lipid accumulation product as a predictor of non-alcoholic fatty liver disease in subjects with different levels of obesity. (DOCX 18 kb)

## Abbreviations

ALT: Alanine transaminase
AUC: Areas under the curves
BMI: Body mass index
BP: Blood pressure
CIs: Confidence intervals
FBG: Fasting blood glucose
HDL-C: High-density lipoprotein cholesterol
LAP: Lipid accumulation product
NAFLD: Non-alcoholic fatty liver disease
ORs: Odds ratios
ROC: Receiver operating characteristic
TBIL: Total bilirubin
TC: Total cholesterol
TG: Triglyceride
WC: Waist circumference

## References

[1] Younossi ZM, Koenig AB, Abdelatif D, Fazel Y, Henry L, Wymer M (2016). Global epidemiology of nonalcoholic fatty liver disease-meta-analytic assessment of prevalence, incidence, and outcomes. Hepatology.

[2] Fan JG (2013). Epidemiology of alcoholic and nonalcoholic fatty liver disease in China. J Gastroenterol Hepatol.

[3] Li Z, Xue J, Chen P, Chen L, Yan S, Liu L (2014). Prevalence of nonalcoholic fatty liver disease in mainland of China: a meta-analysis of published studies. J Gastroenterol Hepatol.

[4] Yu J, Shen J, Sun TT, Zhang X, Wong N (2013). Obesity, insulin resistance, NASH and hepatocellular carcinoma. Semin Cancer Biol.

[5] Wong RJ, Aguilar M, Cheung R, Perumpail RB, Harrison SA, Younossi ZM, Ahmed A (2015). Nonalcoholic steatohepatitis is the second leading etiology of liver disease among adults awaiting liver transplantation in the United States. Gastroenterology.

[6] Fukuda T, Hamaguchi M, Kojima T, Hashimoto Y, Ohbora A, Kato T, Nakamura N, Fukui M (2016). The impact of non-alcoholic fatty liver disease on incident type 2 diabetes mellitus in non-overweight individuals. Liver Int.

[7] Ballestri S, Zona S, Targher G, Romagnoli D, Baldelli E, Nascimbeni F, Roverato A, Guaraldi G, Lonardo A (2016). Nonalcoholic fatty liver disease is associated with an almost twofold increased risk of incident type 2 diabetes and metabolic syndrome. Evidence from a systematic review and meta-analysis. J Gastroenterol Hepatol.

[8] Musso G, Gambino R, Tabibian JH, Ekstedt M, Kechagias S, Hamaguchi M, Hultcrantz R, Hagstrom H, Yoon SK, Charatcharoenwitthaya P (2014). Association of non-alcoholic fatty liver disease with chronic kidney disease: a systematic review and meta-analysis. PLoS Med.

[9] Targher G, Byrne CD, Lonardo A, Zoppini G, Barbui C (2016). Non-alcoholic fatty liver disease and risk of incident cardiovascular disease: a meta-analysis. J Hepatol.

[10] Chalasani N, Younossi Z, Lavine JE, Diehl AM, Brunt EM, Cusi K, Charlton M, Sanyal AJ (2012). The diagnosis and management of non-alcoholic fatty liver disease: practice guideline by the American Association for the Study of Liver Diseases, American College of Gastroenterology, and the American Gastroenterological Association. Hepatology.

[11] Sumida Y, Nakajima A, Itoh Y (2014). Limitations of liver biopsy and non-invasive diagnostic tests for the diagnosis of nonalcoholic fatty liver disease/nonalcoholic steatohepatitis. World J Gastroenterol.

[12] Kahn HS (2005). The "lipid accumulation product" performs better than the body mass index for recognizing cardiovascular risk: a population-based comparison. BMC Cardiovasc Disord.

[13] Bozorgmanesh M, Hadaegh F, Azizi F (2010). Diabetes prediction, lipid accumulation product, and adiposity measures; 6-year follow-up: Tehran lipid and glucose study. Lipids Health Dis.

[14] Motamed N, Razmjou S, Hemmasi G, Maadi M, Zamani F (2016). Lipid accumulation product and metabolic syndrome: a population-based study in northern Iran. Amol J Endocrinol Invest.

[15] Taverna MJ, Martinez-Larrad MT, Frechtel GD, Serrano-Rios M (2011). Lipid accumulation product: a powerful marker of metabolic syndrome in healthy population. Eur J Endocrinol.

[16] Xia C, Li R, Zhang S, Gong L, Ren W, Wang Z, Li Q (2012). Lipid accumulation product is a powerful index for recognizing insulin resistance in non-diabetic individuals. Eur J Clin Nutr.

[17] Bozorgmanesh M, Hadaegh F, Azizi F (2010). Predictive performances of lipid accumulation product vs. adiposity measures for cardiovascular diseases and all-cause mortality, 8.6-year follow-up: Tehran lipid and glucose study. Lipids Health Dis.

[18] Hosseinpanah F, Barzin M, Mirbolouk M, Abtahi H, Cheraghi L, Azizi F (2016). Lipid accumulation product and incident cardiovascular events in a normal weight population: Tehran lipid and glucose study. Eur J Prev Cardiol.

[19] Zhong C, Xia W, Zhong X, Xu T, Li H, Zhang M, Wang A, Xu T, Sun Y, Zhang Y (2016). Lipid accumulation product and hypertension related to stroke: a 9.2-year prospective study among Mongolians in China. J Atheroscler Thromb.

[20] Xiang M, Wang PX, Wang AB, Zhang XJ, Zhang Y, Zhang P, Mei FH, Chen MH, Li H (2016). Targeting hepatic TRAF1-ASK1 signaling to improve inflammation, insulin resistance, and hepatic steatosis. J Hepatol.

[21] Farrell GC, Chitturi S, Lau GK, Sollano JD (2007). Asia-Pacific working party on N. Guidelines for the assessment and management of non-alcoholic fatty liver disease in the Asia-Pacific region: executive summary. J Gastroenterol Hepatol.

[22] Lee YJ, Lee HR, Shim JY, Moon BS, Lee JH, Kim JK (2010). Relationship between white blood cell count and nonalcoholic fatty liver disease. Dig Liver Dis.

[23] Wang J, Xu C, Xun Y, Lu Z, Shi J, Yu C, Li Y (2015). ZJU index: a novel model for predicting nonalcoholic fatty liver disease in a Chinese population. Sci Rep.

[24] Dai H, Lu S, Tang X, Lu M, Chen R, Chen Z, Yang P, Liu C, Zhou H, Lu Y, Yuan H (2016). Combined Association of Serum Uric Acid and Metabolic Syndrome with chronic kidney disease in hypertensive patients. Kidney Blood Press Res.

[25] Dai H, Wang W, Tang X, Chen R, Chen Z, Lu Y, Yuan H (2016). Association between homocysteine and non-alcoholic fatty liver disease in Chinese adults: a cross-sectional study. Nutr J.

[26] Chiang JK, Koo M (2012). Lipid accumulation product: a simple and accurate index for predicting metabolic syndrome in Taiwanese people aged 50 and over. BMC Cardiovasc Disord.

[27] Fan JG, Jia JD, Li YM, Wang BY, Lu LG, Shi JP, Chan LY (2011). Chinese Association for the Study of liver D. Guidelines for the diagnosis and management of nonalcoholic fatty liver disease: update 2010: (published in Chinese on Chinese journal of Hepatology 2010; 18:163-166). J Dig Dis.

[28] Sirota JC, McFann K, Targher G, Johnson RJ, Chonchol M, Jalal DI (2013). Elevated serum uric acid levels are associated with non-alcoholic fatty liver disease independently of metabolic syndrome features in the United States: liver ultrasound data from the National Health and nutrition examination survey. Metabolism.

[29] Morena M, Dupuy AM, Jaussent I, Vernhet H, Gahide G, Klouche K, Bargnoux AS, Delcourt C, Canaud B, Cristol JP (2009). A cut-off value of plasma osteoprotegerin level may predict the presence of coronary artery calcifications in chronic kidney disease patients. Nephrol Dial Transplant.

[30] Hanley JA, McNeil BJ (1983). A method of comparing the areas under receiver operating characteristic curves derived from the same cases. Radiology.

[31] Caldwell S, Argo C (2010). The natural history of non-alcoholic fatty liver disease. Dig Dis.

[32] Lazo M, Hernaez R, Eberhardt MS, Bonekamp S, Kamel I, Guallar E, Koteish A, Brancati FL, Clark JM (2013). Prevalence of nonalcoholic fatty liver disease in the United States: the third National Health and nutrition examination survey, 1988-1994. Am J Epidemiol.

[33] Williams CD, Stengel J, Asike MI, Torres DM, Shaw J, Contreras M, Landt CL, Harrison SA (2011). Prevalence of nonalcoholic fatty liver disease and nonalcoholic steatohepatitis among a largely middle-aged population utilizing ultrasound and liver biopsy: a prospective study. Gastroenterology.

[34] Loguercio C, De Girolamo V, de Sio I, Tuccillo C, Ascione A, Baldi F, Budillon G, Cimino L, Di Carlo A, Di Marino MP (2001). Non-alcoholic fatty liver disease in an area of southern Italy: main clinical, histological, and pathophysiological aspects. J Hepatol.

[35] Bedogni G, Miglioli L, Masutti F, Tiribelli C, Marchesini G, Bellentani S (2005). Prevalence of and risk factors for nonalcoholic fatty liver disease: the Dionysos nutrition and liver study. Hepatology.

[36] Blachier M, Leleu H, Peck-Radosavljevic M, Valla DC, Roudot-Thoraval F (2013). The burden of liver disease in Europe: a review of available epidemiological data. J Hepatol.

[37] Soresi M, Noto D, Cefalu AB, Martini S, Vigna GB, Fonda M, Manzato E, Cattin L, Fellin R, Averna MR (2013). Nonalcoholic fatty liver and metabolic syndrome in Italy: results from a multicentric study of the Italian arteriosclerosis society. Acta Diabetol.

[38] Fazel Y, Koenig AB, Sayiner M, Goodman ZD, Younossi ZM (2016). Epidemiology and natural history of non-alcoholic fatty liver disease. Metabolism.

[39] Xiang S, Hua F, Chen L, Tang Y, Jiang X, Liu Z (2013). Lipid accumulation product is related to metabolic syndrome in women with polycystic ovary syndrome. Exp Clin Endocrinol Diabetes.

[40] Chan L, Xue H, Xiaoya Z, Jiajia X, Wei R, Linman L, Qing L, Lan L (2016). Lipid accumulation product: a simple and accurate index for predicting metabolic syndrome in patients with adult growth hormone deficiency. Exp Clin Endocrinol Diabetes.

[41] Larter CZ, Farrell GC (2006). Insulin resistance, adiponectin, cytokines in NASH: which is the best target to treat?. J Hepatol.

[42] Tilg H, Moschen AR (2006). Adipocytokines: mediators linking adipose tissue, inflammation and immunity. Nat Rev Immunol.

[43] Scorletti E, Byrne CD (2013). Omega-3 fatty acids, hepatic lipid metabolism, and nonalcoholic fatty liver disease. Annu Rev Nutr.

[44] Kobyliak N, Falalyeyeva T, Bodnar P, Beregova T. Probiotics Supplemented with Omega-3 Fatty Acids are More Effective for Hepatic Steatosis Reduction in an Animal Model of Obesity. Probiotics Antimicrob Proteins. 2017;9:123–30. 10.1007/s12602-016-9230-1 27660157

[45] Siddiqui MS, Patidar KR, Boyett S, Smith PG, Sanyal AJ, Sterling RK (2015). Validation of noninvasive methods for detecting hepatic steatosis in patients with human immunodeficiency virus infection. Clin Gastroenterol Hepatol.

[46] Zhang Z, Wang G, Kang K, Wu G, Wang P (2016). Diagnostic accuracy and clinical utility of a new noninvasive index for hepatic steatosis in patients with hepatitis B virus infection. Sci Rep.

[47] Macut D, Tziomalos K, Bozic-Antic I, Bjekic-Macut J, Katsikis I, Papadakis E, Andric Z, Panidis D (2016). Non-alcoholic fatty liver disease is associated with insulin resistance and lipid accumulation product in women with polycystic ovary syndrome. Hum Reprod.

[48] Xu C, Yu C, Ma H, Xu L, Miao M, Li Y (2013). Prevalence and risk factors for the development of nonalcoholic fatty liver disease in a nonobese Chinese population: the Zhejiang Zhenhai study. Am J Gastroenterol.

