# Supplementary material for: Lipid accumulation product is a powerful tool to predict non-alcoholic fatty liver disease in Chinese adults
Source: Nutr Metab (Lond). 2017 Aug 1;14:49. doi: 10.1186/s12986-017-0206-2 (PMC5539973; doi:10.1186/s12986-017-0206-2)
Supplement: Additional file 1: Table S1. — Areas under the ROC curves for lipid accumulation product as a predictor of non-alcoholic fatty liver disease in subjects with different levels of obesity. (DOCX 18 kb) [file 12986_2017_206_MOESM1_ESM.docx]

Additional file 1: Table S1. Areas under the ROC curves for lipid accumulation product as a predictor of non-alcoholic fatty liver disease in subjects with different levels of obesity.

| BMI, kg/m^2^ | Men  *N* | AUC (95% CI) | *P* value | *P* value^#^ | Women  *N* | AUC (95% CI) | *P* value | *P* value^#^ |
| --- | --- | --- | --- | --- | --- | --- | --- | --- |
| ≤22.9 | 5313 | 0.831 (0.813, 0.849) | <0.001 | ─ | 13448 | 0.873 (0.859, 0.887) | <0.001 | ─ |
| 23.0 ~ 24.9 | 4670 | 0.722 (0.707, 0.737) | <0.001 | <0.001 | 4490 | 0.752 (0.736, 0.768) | <0.001 | <0.001 |
| 25.0 ~ 29.9 | 7455 | 0.720 (0.708, 0.733) | <0.001 | <0.001 | 3732 | 0.710 (0.693, 0.727) | <0.001 | <0.001 |
| 30.0 ~ | 869 | 0.706 (0.632, 0.781) | <0.001 | 0.002 | 392 | 0.756 (0.692, 0.819) | <0.001 | <0.001 |

#: comparison of AUC, with BMI ≤22.9 kg/m^2^ serving as the reference.

90 subjects were excluded from this analysis due to BMI data missing.
